# Reliability of Judging in DanceSport

**DOI:** 10.3389/fpsyg.2019.01001

**Published:** 2019-05-07

**Authors:** Jerneja Premelč, Goran Vučković, Nic James, Bojan Leskošek

**Affiliations:** ^1^Faculty of Sport, University of Ljubljana, Ljubljana, Slovenia; ^2^School of Science and Technology, Middlesex University, London, United Kingdom

**Keywords:** DanceSport, ballroom dance, judging system, reliability, validity, aesthetic sports

## Abstract

**Purpose:**

The aim of this study was to assess the reliability and validity of the new judging system in DanceSport.

**Methods:**

Eighteen judges rated the 12 best placed adult dancing couples competing at an international competition. They marked each couple on all judging criteria on a 10 level scale. Absolute agreement and consistency of judging were calculated for all main judging criteria and sub-criteria.

**Results:**

A mean correlation of overall judging marks was 0.48. Kendall’s coefficient of concordance for overall marks (*W* = 0.58) suggesting relatively low agreement among judges. Slightly lower coefficients were found for the artistic part [Partnering skills (*W* = 0.45) and Choreography and performance (*W* = 0.49)] compared to the technical part [Technical qualities (*W* = 0.56) and Movement to music (*W* = 0.54)]. ICC for overall criteria was low for absolute agreement [ICC(2,3) = 0.62] but higher for consistency [ICC(3,3) = 0.80].

**Conclusion:**

The relatively large differences between judges’ marks suggest that judges either disagreed to some extent on the quality of the dancing or used the judging scale in different ways. The biggest concern was standard error of measurement (SEM) which was often larger than the difference between dancers scores suggesting that this judging system lacks validity. This was the first research to assess judging in DanceSport and offers suggestions to potentially improve both its objectivity and validity in the future.

## Introduction

DanceSport consists of three different disciplines: Standard dances (Waltz, Tango, Viennese Waltz, Slow Foxtrot, and Quickstep), Latin-American dances (Samba, Cha-Cha-Cha, Rumba, Paso Doble, and Jive) and Ten Dances (five Standard and five Latin-American dances). A dancer’s success is determined by technical and tactical skills (Uznović and Kostić, 2005; Howard, 2007; Uznović, 2008; Laird, 2009) morphological and motor abilities (Koutedakis, 2008; Lukić et al., 2011; Prosen et al., 2013), psychological preparation and aesthetics of movement (Lukić et al., 2009; Čačković et al., 2012). Furthermore, efficiency in DanceSport has been suggested as a determining factor for a judge to award marks for the dancers’ performance (Bijster, 2013a, 2013b). This is pertinent since judges have a big influence on the rules, judging and, of course, the final result for a dance performance. Judging in DanceSport is characterized by a subjective marking system that is often criticized because of lack of objectivity. Judges are responsible for quickly and accurately discerning the quality of technical elements and overall aesthetic appearance of a dancer’s performance based upon their perception

of the performance. To make it harder they need to evaluate six or twelve couples on the dance floor in just a minute and a half.

Prior to the introduction of a new judging system in 2013, the judging system in DanceSport had not been changed for many years, in contrast to many other aesthetic sports like gymnastic, figure-skating, etc., where changes had been made during the last decade (Boen et al., 2008; Dallas and Kirialanis, 2010). There have been some criticisms of the old judging system in DanceSport by dancers, coaches, and judges with unsubstantiated suggestions that some dancers were favored over others, there was insufficient time to properly evaluate each dancer and that dancers did not get sufficient feedback on the quality of their performance (Ambrož, 2010; Bijster, 2012; Hurley, 2012; Malitowska, 2013). Whilst these comments are hearsay, the World DanceSport Federation did construct a new judging system using a similar model to figure-skating and presented it in September 2013. The purpose of this new system was, theoretically, to allow more objective and reliable judging and to give better feedback to dancers in regard to specific criteria of their performance. The main differences of the new system are the determination of four main judging criteria, a higher number of judges used and a lower number of dancers dancing at the same time. Dancers perform three dances solo and two dances with six couples on the dance floor at the same time.

Several aspects of judging performance in aesthetic sports have been described (Popović, 2000; Plessner and Schallies, 2005; Leskošek et al., 2010; Bučar Pajek et al., 2011). Studies have shown that changes in the judging system have usually resulted in higher objectivity of judging (Lockwood et al., 2005; Atiković et al., 2011; Leskošek et al., 2013). To date, there is a lack of studies for judging in dance, with consequent concern for the possibility of systematic bias and inconsistency of judging in DanceSport, which could influence competition results. Research into the quality of judging is therefore seen as a necessity. We therefore designed this study to assess the reliability and validity of the new judging system in DanceSport by measuring the agreement and consistency between judges for all criteria.

## Materials and Methods

### Ethics Statement

This study was approved by the Ethics Committee of the Faculty of Sport at the University of Ljubljana. The study’s objectives and methods were explained to each participant, before a written, informed consent was obtained.

### Participants

Eighteen judges, two national and 16 WDSF international licensed, had an average judging experience of 19.6 ± 9 years. All judges were educated on the new judging system and had participated in seminars and undertaken the annual judging exam. All of them had previously competed as sport-dancers and most were still coaches.

### Procedure

The new judging system consists of technical and artistic parts which each have two criteria, the content of which is defined in Table 1. Judging involves four groups of three judges, each group judging only one criterion, which is randomly selected. Each of the three judges in each group therefore judge the dancers on the four sub-criteria but only award one mark which is the main criterion score. These three scores are then put into a formula to calculate the final mark awarded for the main criterion. The judging scale offers 10 different quality rating levels, with 0.5 subdivisions (total of 21 points of evaluation). A description of performance is defined for each level (0–10). Dancers perform three dances solo and two dances with six couples on the dance floor at the same time.

**TABLE 1:** Judging criteria issued by the World DanceSport Federation (2013).

**Technical part**		**Artistic part**	
**Technical qualities**	**Movement to music**	**Partnering Skills**	**Choreography and**
			**performance**
Posture and hold	Timing	Couple position	Choreography
General principles	Shuffle timing	Leading	Creativity, personal style
Basic actions	Specific rhythm requirements	Basic action – partnering	Expression, interpretation
Specific principles	Personal interpretation	Line Figures – partnering	Characterization

For this study judges rated the 12 best placed adult dancing couples (over 19 years of age) competing at an international competition, the International Open 2012 in Ljubljana, Slovenia. Judges viewed the dances from video (filmed from the same position as judges would be standing at the competition) projected onto a big screen. Judges had 1 min to mark one couple on all four sub-criteria for one main criterion on a 10 levels scale, as would be the case for the real competition. However, whereas in a competition a judge would not need to give a mark for each sub-criteria, i.e., only the main criterion, in this study they were asked to award marks for all sub-criteria also. The order of judging criteria and dancing couples were randomly selected for each judge.

### Data Analysis

Basic distributional parameters and Pearson *r* correlation coefficient were computed for all marks for each individual judge. A Total mark was also computed as the mean of the marks for the four main criteria. Inter-rater reliability was evaluated by *MeanCor* (average *r* between all 18 judges in each main and sub-criteria), Kendall’s *W* coefficient of concordance and ICCs (intra-class correlation coefficients). Using the notation of Shrout and Fleiss (1979) the following ICCs were computed: ICC(2,1), ICC(2,3), ICC(3,1), and ICC(3,3), i.e., single and average measures for both two-way random (consistency) and fixed (agreement) effects. ICC(2,3) and ICC(3,3) were computed from ICC(2,1) and ICC(3,1) using Spearman–Brown prediction formula. Additionally, SEM (standard error of measurement) was calculated as SD*[1–ICC(2,3)]^1/2^, where SD is standard deviation of competitors scores for a criterion. All calculations were performed with *irr* and *psych* libraries of R software (The R project for statistical computing, 2015).

## Results

**FIGURE 1:**
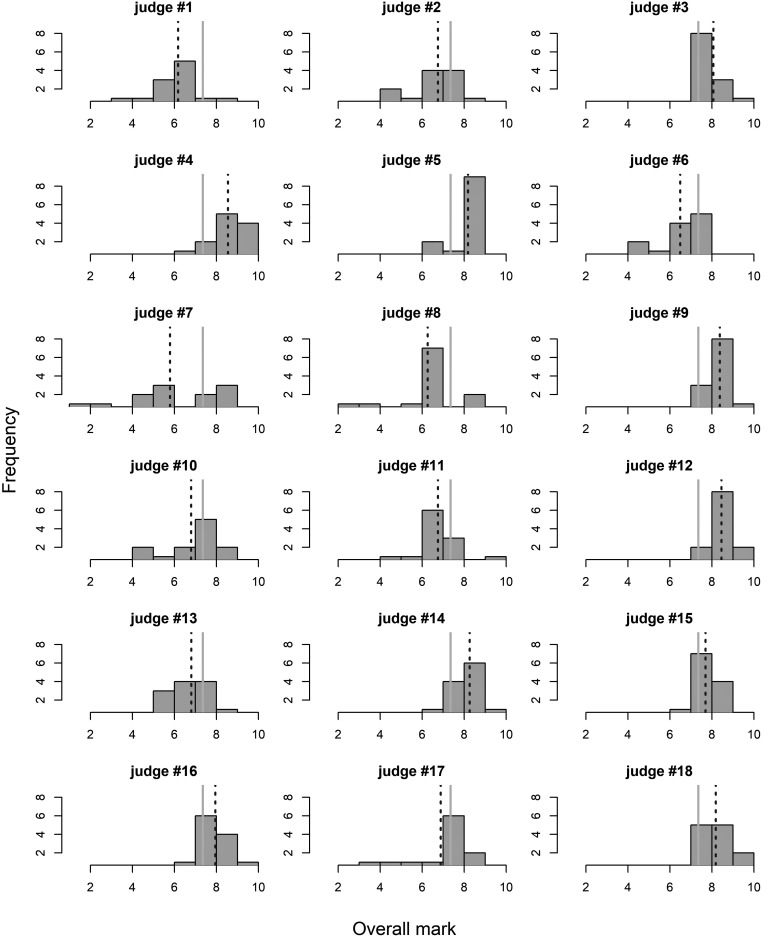
Histograms of the overall marks for competitors (*n* = 12) by individual judges. Key: solid vertical lines denote grand average (all judges combined), while dotted lines denote averages for individual junges.

Overall marks (average of marks for technical qualities, movement to music, partnering skills and choreography presentation) showed great variability among judges (Figure 1). Range widths varied from less than two points (judges #3 and #9) to more than six points (judges #7 and #8). Individual judge’s averages varied between being 1.57 points (judge #7) below the average of all judges to being more than 1 point (judges #4, #9 and #12) above. Minimum marks for individual judges were, in most cases, above 4 points (in half of the judges above 6 points), but as low as 1.25 point for judge #7. Similarly, maximum marks vary from 7.75 (judge #6) to 9.50 (judges #4, #12, and #18). Also extreme difference between judges exist in variability of their marks, e.g., between judge #3 (standard deviation *s* = 0.51, coefficient of variation CV = 0.06), and judge #7 (*s* = 2.30, CV = 0.40).

Combining the judges scores resulted in a mean value for the overall mark of 7.33 with mean standard deviation 1.22 (Table 2). However, the difference between the minimum and maximum of the lowest and the highest marks was 6.5 and 1.5 points, respectively. Overall judging marks varied for the mean (5.77–8.53) and *s* (0.67–2.42) with larger differences for mean values for Partnering skills (*M* = 5.67–8.7) than Choreography and performance (*M* = 5.53–8.54), Movement to music (*M* = 5.92–8.71), and Technical qualities (*M* = 5.92–8.5).

**TABLE 2:** Mean, average, minimum and maximum values for judging criteria.

**Judging criteria**	**Mean values**			**Standard deviation**			**The lowest marks**			**The highest marks**		
	**M**	**min**	**max**	**M**	**min**	**max**	**M**	**min**	**max**	**M**	**min**	**max**
**Technical qualities**	**7.36**	**5.93**	**8.5**	**1.15**	**0.7**	**2.1**	**5.25**	**1.0**	**7.5**	**8.89**	**8.0**	**9.5**
TQ1 – Posture and hold	7.47	6.04	8.54	1.11	0.66	2.3	5.31	0.5	7.5	8.97	8.5	9.5
TQ2 – General principles	7.35	5.79	8.5	1.21	0.68	2.41	5.08	0.5	7.0	8.94	8.0	9.5
TQ3 – Basic actions	7.31	5.88	8.58	1.18	0.63	2.01	5.14	1.0	7.5	8.97	8.0	9.5
TQ4 – Specific principles	7.23	5.67	8.5	1.2	0.54	2.06	5.08	1.5	7.5	8.89	7.5	9.5
**Movement to music**	**7.34**	**5.92**	**8.71**	**1.38**	**0.54**	**3.01**	**4.83**	**1.0**	**7.5**	**9.03**	**8.0**	**9.5**
MM1 – Timing	7.56	6.08	8.67	1.29	0.62	2.9	5.11	1.0	7.5	9.17	8.0	9.5
MM2 – Shuffle timing	7.27	5.79	8.63	1.45	0.65	3.06	4.69	1.0	7.5	9.14	8.0	9.5
MM3 – Specific rhythm requirements	7.28	5.67	8.75	1.35	0.54	2.76	4.81	1.0	7.5	8.97	8.0	9.5
MM4 – Personal interpretation	7.17	5.5	8.58	1.53	0.67	3.4	4.47	0.5	7.5	9.14	8.0	10.0
**Partnering skills**	**7.37**	**5.67**	**8.71**	**1.15**	**0.53**	**2.16**	**5.39**	**1.5**	**7.5**	**8.94**	**7.5**	**9.5**
PS1 – Couple position	7.52	5.92	8.63	1.03	0.48	1.84	5.81	2.0	7.5	9.03	8.0	9.5
PS2 - Leading	7.42	5.63	8.75	1.24	0.52	2.54	5.28	1.0	7.5	9.06	8.0	9.5
PS3 – Basic action – partnering	7.3	5.42	8.71	1.16	0.61	2.17	5.22	2.0	7.5	8.94	7.5	9.5
PS4 – Line Figures – partnering	7.21	5.58	8.58	1.26	0.62	2.8	5.06	0.5	7.5	8.97	7.5	9.5
**Choreography and performance**	**7.35**	**5.53**	**8.54**	**1.08**	**0.45**	**2.41**	**5.5**	**1.5**	**7.5**	**8.86**	**7.5**	**9.5**
CP1 - Choreography	7.48	5.71	8.63	1.02	0.42	2.35	5.67	1.5	8.0	8.92	8.0	9.5
CP2 – Creativity. Personal style	7.15	5.17	8.54	1.17	0.5	2.59	5.14	1.0	7.5	8.86	7.0	9.5
CP3 – Expression. Interpretation	7.23	5.71	8.46	1.16	0.45	2.35	5.31	3.0	7.5	8.92	7.5	9.5
CP4 - Characterization	7.29	5.46	8.54	1.17	0.48	2.78	5.31	1.0	7.5	8.97	8.0	9.5
**Overall marks**	**7.33**	**5.77**	**8.53**	**1.22**	**0.67**	**2.42**	**4.00**	**0.5**	**7.0**	**9.36**	**8.5**	**10.0**

A mean correlation of overall judging marks was 0.48 (Table 3) with correlations ranging between Partnering skills (*M* = 0.46) and Choreography and performance (*M* = 0.49) to Movement to music (*M* = 0.59) and Technical qualities (*M* = 0.60). However, correlation coefficients ranged between 0.06 and 0.84. For the reliability of judging the Kendall’s W coefficient and ICC of a single (x,1) and three (x,3) judges (as used in the new system) were considered. Kendall’s coefficient of concordance for overall marks (*W* = 0.58) suggested low agreement among judges. Slightly lower coefficients were found for the artistic part [Partnering skills (*W* = 0.45) and Choreography and performance (*W* = 0.49)] compared to the technical part [Technical qualities (*W* = 0.56) and Movement to music (*W* = 0.54)]. The lowest value was for the sub-criterion Leading (*W* = 0.41). Similar results were found for ICC coefficients with the ICC for overall criteria low for absolute agreement [ICC(2,3) = 0.62] but higher for consistency [ICC(3,3) = 0.80].

**TABLE 3:** Reliability of judging of all main and sub-criteria.

**Judging criteria**	**Correlation**			**Kendall’s W**	**ICC coefficient**				**SEM^#^**
				**coefficient**					
					**Absolute agreement**		**Consistency (C)**		
					**(AA) (number of judges)**		**(number of judges)**		
	
	**Mean**	**Min**	**Max**	Value*	**1**	**3**	**1**	**3**	**3**
**Technical qualities**	**0.60**	**0.24**	**0.83**	**0.56**	**0.37**	**0.63**	**0.55**	**0.78**	**0.56**
TQ1–Posture and hold	0.53	0.15	0.80	0.47	0.33	0.60	0.48	0.74	0.54
TQ2–General principles	0.54	0.17	0.83	0.52	0.34	0.60	0.50	0.75	0.59
TQ3–Basic actions	0.60	0.22	0.81	0.57	0.36	0.63	0.55	0.79	0.58
TQ4–Specific principles	0.53	0.2	0.80	0.53	0.30	0.57	0.48	0.73	0.60
**Movement to music**	**0.59**	**0.24**	**0.83**	**0.54**	**0.37**	**0.64**	**0.50**	**0.75**	**0.67**
MM1–Timing	0.55	0.16	0.80	0.52	0.38	0.65	0.48	0.73	0.61
MM2–Shuffle timing	0.60	0.30	0.82	0.57	0.36	0.63	0.48	0.74	0.70
MM3–Specific rhythm requirements	0.61	0.28	0.84	0.57	0.37	0.64	0.51	0.76	0.67
MM4–Personal interpretation	0.60	0.28	0.83	0.55	0.36	0.63	0.49	0.75	0.76
**Partnering skills**	**0.46**	**0.07**	**0.75**	**0.45**	**0.25**	**0.50**	**0.38**	**0.65**	**0.57**
PS1–Couple position	0.46	0.09	0.78	0.45	0.24	0.49	0.38	0.64	0.52
PS2–Leading	0.41	0.22	0.74	0.41	0.24	0.48	0.35	0.62	0.61
PS3–Basic action–partnering	0.45	0.03	0.72	0.44	0.23	0.47	0.37	0.64	0.59
PS4–Line figures–partnering	0.44	0.02	0.76	0.43	0.25	0.50	0.36	0.63	0.63
**Choreography and performance**	**0.49**	**0.00**	**0.79**	**0.49**	**0.26**	**0.52**	**0.41**	**0.68**	**0.54**
CP1–Choreography	0.42	−0.06	0.76	0.44	0.24	0.48	0.36	0.63	0.50
CP2–Creativity, personal style	0.46	−0.02	0.76	0.45	0.23	0.47	0.39	0.66	0.60
CP3–Expression, interpretation	0.45	−0.01	0.77	0.44	0.26	0.51	0.39	0.65	0.57
CP4–Characterization	0.44	−0.05	0.74	0.46	0.25	0.50	0.37	0.64	0.58
**Overall marks**	**0.48**	**0.21**	**0.65**	**0.58**	**0.36**	**0.62**	**0.57**	**0.80**	**0.54**

*All Kendall’s W coefficients were statistical significant at *p* < 0.01. #SEM – standard error of measurement [also called typical error, see Hopkins (2000)].

Reliability was also estimated separately for the order in which judges evaluated the sub-criteria. Average ICC(1,3) for the 20 criteria were 0.50, 0.56, 0.59, and 0.51, respectively.

**FIGURE 2:**
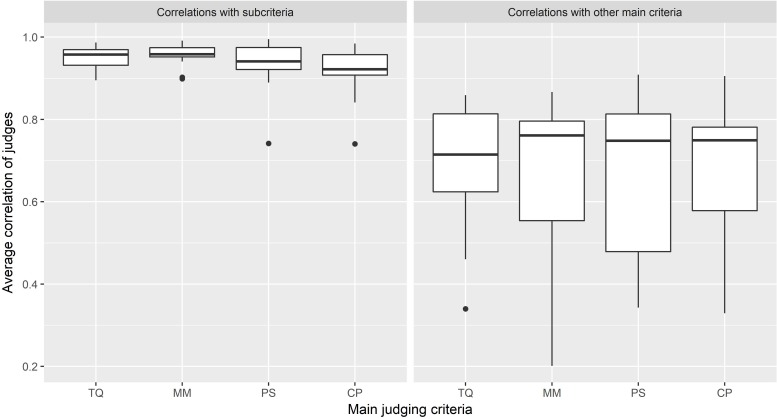
Average correlations between each of the main criteria and it’s sub-criteria and between each of the main criteria and other main criteria by judges.

All of the judges’ marks for the four main criteria were highly correlated with their own sub-criteria (Figure 2). However, the correlations between the four main criteria were much lower, especially for some judges (#3, #13, and #15).

## Discussion

The relatively large differences between judges’ marks (mean values ranged between 5.77 and 8.53) suggest differences in how judges perceived the quality of the dancers or their interpretation of the judging scale. The use of three judges in the new judging system helps reduce inter-judge differences by averaging, thus reducing the effect of extreme scores, evident in the ICC coefficients for three judges compared to for one. The ICC coefficients and Kendall’s coefficients of concordance were, however, quite low suggesting poor validity of the new judging system. Since dancing experts, judges, coaches, and dancers contributed to this system a potential solution could be to learn from other sports which have changed their judging systems in a positive way (Boen et al., 2008; Dallas and Kirialanis, 2010; Leskošek et al., 2010).

The relatively high standard errors of measurement (SEM), e.g., 0.54 for the overall marks was higher than the difference between scores for consecutively ranked dance couples in all but one case (0.99 between places 10 and 11) and higher than the difference between “bronze medal” and 9th position. As SEM is only one part of MD (*minimum difference to be considered real*, usually computed as SEM*1.96*2^1/2^; Weir, 2005), it seems that the rank order of pairs is not defensible as the error of measurement is in many cases much higher than the actual differences between pairs.

The present judging scale offers 10 different quality rating levels. Whilst a description of performance is defined for each level (0–10) it may be the case that judges do not adopt these criteria easily (hence the differences in scores) and subconsciously create their own marking scale and continue to rank dancing couples against each other, the opposite of the intention of the new judging system. A comparable judging scale (0 to 10) is used in figure-skating, where the description of each level includes the content of the performed elements along with technical and artistic descriptors. The quality of required elements is specifically defined which also includes possible mistakes in a performance (International Skating Union, 2015). Potentially DanceSport should define a similar scale to that used in skating, where descriptors of quality for each criteria and sub-criteria are defined.

The judging of the technical parts of performance was shown to be more reliable than the artistic parts, which could be a result of more detailed criteria for the technical parts. Lockwood et al. (2005) also found that judging the technical part was more reliable than judging the artistic part in ice-skating. Similarly, Pajek et al. (2014) noted that judging the artistry component in gymnastic showed poor reliability among judges. Vermey and Brandt (2002) suggested that judges rely on their knowledge and ability to recognize artistic qualities, allowing them to ascribe value to a performance. Observers may evaluate aesthetic through cognitive judgment or affective appreciation of dance movement, while others may include their own familiarity and physical ability in their aesthetic appreciation (Chatterjee, 2003; Leder et al., 2004; Cross et al., 2011). Torrents et al. (2013) and Neave et al. (2010) suggested that there were strong associations between higher beauty scores and certain kinematic parameters, especially the amplitude of movement. Choreography in DanceSport is undefined and it is unclear what determines good choreography. Adult dancers have a free choice of choreography and judges interpret the quality of choreography seemingly from a personal perspective. In other aesthetic sports the choreography and its elements are precisely evaluated. For example, the rules of gymnastics for all disciplines describe choreography according to difficulty and correct performance (International Gymnastics Federation, 2015). Hence DanceSport should consider adopting similar descriptions for choreography.

The correlations between each of the main criteria with their sub-criteria were generally high suggesting that judging only four main criteria without including the sub-criteria would have little impact on the overall scores. Lower correlations confirm that judges assess the four main criteria separately whilst higher correlations cannot confirm this as judges could either assess the four criteria correctly as the same or are incapable of separating the criteria. Whilst these findings are ambiguous it is recommended that DanceSport considers using only two criteria, one for the technical component and the other artistic, as used in the figure skating judging system. In this case each criterion could have six judges instead of three, which would contribute to a more reliable judging system allowing for the elimination of extreme values.

Although the judging process includes specific objective criteria, judges may still rely on subjective determinations and perceptions. For example, dancing experts are already warning that the new system brings no improvement and is still too subjective (Bijster, 2012). Subjectivity is bound to reduce the reliability of a judging system unless judges are selected for their personal biases. For example an choreographer might tend to give dancers with excellent choreography higher marks than perhaps the other elements of the dance deserves (Vermey, 1994). Findlay and Ste-Marie (2004) also suggested that subjectivity in rating also biases toward athletes with high reputations. There are also other factors that can potentially influence judging. Ste-Marie (1999) found that judge’s education and experience significantly contributed to the evaluation of ice-skater’s performance. Fernandez-Villarino et al. (2013) noted that the most valued abilities by the judges are knowledge of the technical parameters of the sport and the capacity to adjust to any level of competition with self-assuredness and self-confidence. Some studies have uncovered order effects, pursuant to which competitors who appear later in a sequence of performances tend to receive higher scores than those who appear earlier (Greenlees et al., 2007). Bruine de Bruin (2006), found that figure skaters who performed later in the first round received better scores in the first and second round. This may be because judges feel uncertain about how to judge the first few performers and to be safe, initially use scores from the middle of the scale, saving more extreme scores for later contestants (Bruine de Bruin, 2005).

A problem or determining an accurate judging system for DanceSport also occurs because there are two organizations overseeing this process. At present the WDC does not use the new judging system whereas the WDSF does with the goal of improving judging to a level acceptable for DanceSport becoming an Olympic sport in 2020. It would seem advisable that these two bodies come together to harmonize opinion as to what constitutes quality in DanceSport.

Whilst this study attempted to provide optimal and comparable conditions to the real competition setting, a limitation of this research was that judges evaluated dancing couples by watching a videotape on a big screen. Whilst they had the same viewing position as the judges had standing at the competition, the unfamiliar surroundings may have had some impact on their judging. They also only judged the top 12 placed dancing couples, because they were the only ones to undertake solo dances. This allowed the judges a better view of each couple compared to if there had been 6 or 12 couples dancing at the same time. However, the lower number of couples and smaller differences in quality between them would tend to lower the intra-class reliability coefficients. It should also be noted that the judges in this study viewed videos of the same couples four times (to judge each main criteria separately), which may have influenced their marking. However, the differences in the reliability for these marks were low suggesting this was not a major concern.

## Conclusion

The new judging system appears to be too subjective, which would account for a lower than possible reliability rating. This could be solved by determining the content and difficulty for each main and sub-criterion more precisely. Independent criterion should be described and the judging scale should have more precise definitions for each level including perfect presentation and presentation with small or major mistakes. If the judging system is rewritten as suggested the new criteria should be made available to all judges, dancers, and coaches.

## Data Availability

All datasets generated for this study are included in the manuscript and/or the supplementary files.

## Ethics Statement

This study was carried out in accordance with the recommendations of the Ethics Committee of the Faculty of Sport at the University of Ljubljana, with written informed consent from all subjects. All subjects gave written informed consent in accordance with the Declaration of Helsinki. The protocol was approved by the Ethics Committee of the Faculty of Sport at the University of Ljubljana.

## Author Contributions

JP and BL made substantial contribution to the conception and study design, data acquisition, analysis, and interpretation. GV contributed to the drafting of the manuscript and revised it critically for important intellectual content. NJ contributed to the final approval version of the manuscript.

## Conflict of Interest Statement

The authors declare that the research was conducted in the absence of any commercial or financial relationships that could be construed as a potential conflict of interest.
